# MCL-1, BCL-X_L_ and MITF Are Diversely Employed in Adaptive Response of Melanoma Cells to Changes in Microenvironment

**DOI:** 10.1371/journal.pone.0128796

**Published:** 2015-06-02

**Authors:** Mariusz L. Hartman, Beata Talar, Anna Gajos-Michniewicz, Malgorzata Czyz

**Affiliations:** Department of Molecular Biology of Cancer, Medical University of Lodz, Lodz, Poland; University of Colorado, School of Medicine, UNITED STATES

## Abstract

Melanoma cells can switch their phenotypes in response to microenvironmental insults. Heterogeneous melanoma populations characterized by long-term growth and a high self-renewal capacity can be obtained *in vitro* in EGF(+)bFGF(+) medium whilst invasive potential of melanoma cells is increased in serum-containing cultures. In the present study, we have shown that originally these patient-derived melanoma populations exhibit variable expression of pro-survival genes from the BCL-2 family and inhibitors of apoptosis (IAPs), and differ in the baseline MCL-1 transcript stability as well. While being transferred to serum-containing medium, melanoma cells are well protected from death. Immediate adaptive response of melanoma cells selectively involves a temporary MCL-1 increase, both at mRNA and protein levels, and BCL-X_L_ can complement MCL-1, especially in MITF_low_ populations. Thus, the extent of MCL-1 and BCL-XL contributions seems to be cell context-dependent. An increase in MCL-1 level results from a transiently enhanced stability of its transcript, but not from altered protein turnover. Inhibition of MCL-1 preceding transfer to serum-containing medium caused the induction of cell death in a subset of melanoma cells, which confirms the involvement of MCL-1 in melanoma cell survival during the rapid alteration of growth conditions. Additionally, immediate response to serum involves the transient increase in MITF expression and inhibition of ERK-1/2 activity. Uncovering the mechanisms of adaptive response to rapid changes in microenvironment may extend our knowledge on melanoma biology, especially at the stage of dissemination.

## Introduction

Phenotypic reprogramming of both normal [1,2] and to a larger extent cancer cells [3–5] enables them to adapt to fluctuating parameters of the microenvironment. Consequently, functional features of cancer cells are not static entities, but rather may be reversibly turned on and off [6–8]. Such a phenotypic instability has been strongly implicated in melanoma biology [9–12]. In response to microenvironmental cues, switching between different cellular programs can be regulated back and forth, and melanoma cells with stem-like characteristics can be generated as well [13]. Therefore, the phenotype of melanoma cells is dynamic rather than defined by intrinsic parameters, questioning the hierarchical organization of melanomas. This is supported by the difficulties to conclusively correlate stem-like features of melanoma cells with any marker [14–18]. At the molecular level, epigenetic mechanisms [19–21] and variable activity of microphthalmia-associated transcription factor (MITF) [13,18,22–27] have been associated with interconversions of melanoma cell phenotypes. Of note, these switches may be used as soon as malignant cells encounter an appropriate microenvironment. Thus, the interaction with the tumor microenvironment could better explain the origin of melanoma cells with diverse programs, including pro-metastatic competence [13], as supported by clinical data showing that the acquisition of the ability to metastasize may occur early in melanoma development [28].

We and others have shown that without any genetic manipulation or selection of a specific subpopulation, but only through small changes in the composition of culture medium, melanoma cells can acquire strikingly different phenotypes [14,21,25,29–31]. Melanoma populations maintained in epidermal growth factor (EGF)- and basic fibroblast growth factor (bFGF)-containing medium (EGF(+)bFGF(+) medium) are enriched with cells possessing stem-like characteristics as exhibited by their high clonogenicity [31], enhanced tumorigenicity [14,29], pluripotency related to increased expression of NANOG and OCT4 [14,21,30] and the ability to differentiate into multiple non-melanocytic lineages [18,29,30]. Melanoma populations grown in EGF(+)bFGF(+) medium may also be endowed with a unique composition of pro-survival machinery as shown for highly up-regulated *BCL2A1* in heterogeneous melanospheres [25]. Importantly, replacement of EGF/bFGF with serum (FBS) induces growth as monolayers, significantly reduces stem-like features including self-renewal capacity [31], enforces proliferation [31] and invasive potential of melanoma cells [25], all preceded by substantial alterations in the gene expression profile [25,30]. Thus, microenvironment-mediated transcriptional reprogramming of melanoma cells may result in long-term phenotypic effects. However, mechanisms underlying the immediate adaptation of melanoma cells to different microenvironment components are poorly elucidated. This is an important issue of melanoma biology as melanoma cells are highly invasive, and intravasation and hematogenous dissemination require well developed protection from cell death and ability of rapid adaptation to the new microenvironment [32].

In the present study, we have used treatment-naïve patient-derived melanoma populations to unravel how melanoma cells exploit survival signals when microenvironment components, particularly growth stimuli EGF and bFGF are replaced by serum.

## Materials and Methods

### Cell Culture

Melanoma cells from untreated patients were obtained during surgical interventions and cultured as described previously [31,33]. They were named DMBC10, DMBC12, DMBC17 and DMBC19 (Department of Molecular Biology of Cancer). This study was approved by the Ethical Commission of the Medical University of Lodz, and written informed consent was obtained from the patients. All populations were continuously grown as anchorage-independent cultures in stem cell medium (named hereafter EGF(+)bFGF(+) medium) consisting of Dulbecco’s Modified Eagle’s Medium (DMEM)/F12 (Lonza, Basel, Switzerland), B-27 supplement (Gibco, Paisley, UK), growth factors (10 ng/ml basic fibroblast growth factor (bFGF) and 20 ng/ml epidermal growth factor (EGF); BD Biosciences, San Jose, California, USA), insulin (10 mg/ml), heparin (1 ng/ml), and antibiotics (100 IU/ml penicillin, 100 mg/ml streptomycin, and 2 mg/ml fungizone B). Twice a week, the medium was exchanged. Every few weeks, single-cell suspensions were generated by enzymatic digestion of melanoma populations. All cultures were dissociated one day prior experiments and were exposed to altered growth conditions as single cells. For that, melanoma cells were transferred from EGF(+)bFGF(+) medium to serum-containing medium in which the growth factors, insulin and heparin were replaced with 10% fetal bovine serum (FBS). The plating density of 1.5^.^ 10^5^ cells/ml was used in all experiments. Cells were collected for analysis at indicated time points. In some control experiments, melanoma cells were exposed in parallel to fresh EGF(+)bFGF(+) medium to exclude effects induced by medium exchange itself. In other control experiments, cells were grown in serum-containing medium for two weeks (long-term effects). An acid phosphatase activity (APA) assay was used to validate the proliferation rate and doubling time as described previously [31]. The doubling time (DT) was calculated using the formula DT = (t—t_o_)log 2/(log A—log A_o_), in which t and t_o_ are the times at which the cells were assessed and A and A_o_ are the absorbance at times t and t_o_, respectively. Normal human adult epidermal melanocytes (NHEM) (Lonza) were grown in MBM-4 medium containing growth factors and supplements: calcium chloride, BPE, rh-FGF-B, rh-insulin, hydrocortisone, PMA, gentamicin/amphotericin-B, endothelin-3 and FBS (Lonza).

### Microscopy

Cell morphology was registered with a digital Olympus camera (C-5050) attached to Olympus microscope (Olympus CKX41; Olympus Optical Co, London, UK). Cells stained with acridine orange and ethidium bromide were analyzed under a fluorescence microscope (Olympus BX41).

### Acridine orange/ethidium bromide staining

The procedure of acridine orange/ethidium bromide staining was described previously [34]. Cells were harvested, trypsinized and washed with PBS. Pellet was resuspended in 100 μg/ml acridine orange and 100 μg/ml ethidium bromide (Sigma-Aldrich, St. Louis, MO, USA) solved in phosphate-buffered saline (PBS). Cells were immediately mounted on the slides and analyzed under a fluorescence microscope. At least 300 cells were counted to determine the percentage of viable and apoptotic/necrotic cells.

### Flow cytometry

Induction of apoptosis was monitored by using Annexin V-FLUOS Staining Kit (Roche Diagnostics, Manheim, Germany) according to the procedure described previously [35]. Cells were harvested, trypsinized and washed with PBS. Pellet was resuspended in Annexin V binding buffer containing FITC-conjugated Annexin V and propidium iodide (PI), and incubated for 15 min at room temperature in the dark. Acquisition of 30,000 events was performed using FACSVerse (BD Biosciences) and data were analyzed with FACSuite software (BD Biosciences).

### RNA isolation, cDNA synthesis and Real-Time PCR (qRT-PCR)

Total RNA was collected and purified using Total RNA Isolation kit as described previously [36]. cDNA was synthesized from 1 μg of total RNA using random primers and SuperScript II Reverse Transcriptase (Invitrogen Life Technologies, Carlsbad, CA, USA). The analysis of transcript levels of selected genes was performed by quantitative real-time polymerase chain reaction (qRT-PCR) by using the Rotor-Gene 3000 Real-Time DNA analysis system (Corbett Research, Morklake, Australia). Amplification was performed by using KAPA SYBR FAST qPCR Kit Universal 2X qPCR Master Mix (Kapa Biosystems, Cape Town, South Africa), 200 nM of each primer and 25 ng cDNA template per reaction. The primer sequences used for qRT-PCR are shown in S1 Table. The annealing temperature for all genes was 56°C. A mathematical model including an efficiency correction for qRT-PCR was used to calculate the relative expression levels of target genes *versus* a reference gene *RPS17*.

### Preparation of cell lysates and Western blotting

Cell lysates were prepared and Western blotting was performed as described previously [35]. Briefly, cells were lysed in RIPA buffer containing freshly added protease and phosphatase inhibitors. Protein concentration was determined by Bradford assay (BioRad, Hercules, CA, USA). Samples were loaded on standard 7% SDS-polyacrylamide gel. The proteins were transferred onto Immobilon-P PVDF membrane (Millipore, Billerica, MA, USA). Then, the membrane was incubated for 1 hour in blocking solution (5% non-fat milk in TBS-Tween 0.05%), or in phosphoBLOCKER (Cell Biolabs, San Diego, CA, USA) in TBS-Tween 0.05% when phosphorylated proteins were immunodetected. Primary antibodies detecting PARP (1:1000, rabbit polyclonal, sc-7150), MCL-1 (1:1000, rabbit polyclonal, sc-20679) (Santa Cruz Biotechnology, Santa Cruz, CA, USA), BCL-X_L_ (1:1000, rabbit monoclonal, #2764), BCL-2 (1:1000, rabbit monoclonal, #2870), ERK-1/2 (1:1000, mouse monoclonal, #9107), p-ERK-1/2 (1:1000, rabbit monoclonal, #4377), ubiquitin (1:1000, mouse monoclonal, #3936) (Cell Signaling Technology, Danvers, MA, USA), or β-actin (1:2500, rabbit polyclonal, A2066) (Sigma-Aldrich) were used followed by binding the secondary HRP-conjugated anti-mouse or anti-rabbit antibodies (Santa Cruz Biotechnology). The proteins were visualized by using Pierce ECL Western Blotting Substrate (Pierce, Rockford, IL, USA). The quantification of the Western blotting data was performed by using ImageJ software.

### Transfection of melanoma cells with siRNAs for MCL-1 and BCL-X_L_

Melanoma cells from EGF(+)bFGF(+) cultures were transfected with siRNAs targeting MCL-1 or BCL-X_L_ (Santa Cruz Biotechnology) by using AMAXA NHEM-Neo Nucleofector Kit and Nucleofector 2b device (Lonza) according to the manufacturer’s protocol. The nucleofection program T-030 was chosen to provide both efficient silencing of MCL-1/BCL-X_L_ expression and high enough cell viability. To evaluate the off-target effects of the siRNAs, cells were transfected with control siRNA-A (Santa Cruz Biotechnology). Cells were incubated for 16 h before they were transferred either to fresh EGF(+)bFGF(+) medium for further culturing or serum-containing medium. After indicated time intervals, cells were used for viability assays (Annexin V/PI and acridine orange/ethidium bromide analyses) and cell lysate preparation.

### mRNA decay assay

Cells grown in EGF(+)bFGF(+) medium or serum-containing medium were incubated with 2 μg/ml actinomycin D (Sigma-Aldrich) to inhibit *de novo* RNA synthesis. Concomitantly, cells were transferred from EGF(+)bFGF(+) to serum-containing medium supplemented with 2 μg/ml actinomycin D. Cells were collected at 1, 2, 4 and 8 h intervals after treatment with actinomycin D for total RNA isolation and qRT-PCR analysis. The half-lives (t_1/2_) of MCL-1 mRNA were calculated and used to compare transcript stabilities.

### Statistical Analysis

In all experiments, the data represent the means ± SD. Student’s t-test was performed in Microsoft Excel to determine significant differences between the mean values of the tested parameters. The difference was considered significant if *P* < 0.05.

## Results

### Morphologic and molecular characteristics of patient-derived melanoma populations

**Fig 1:**
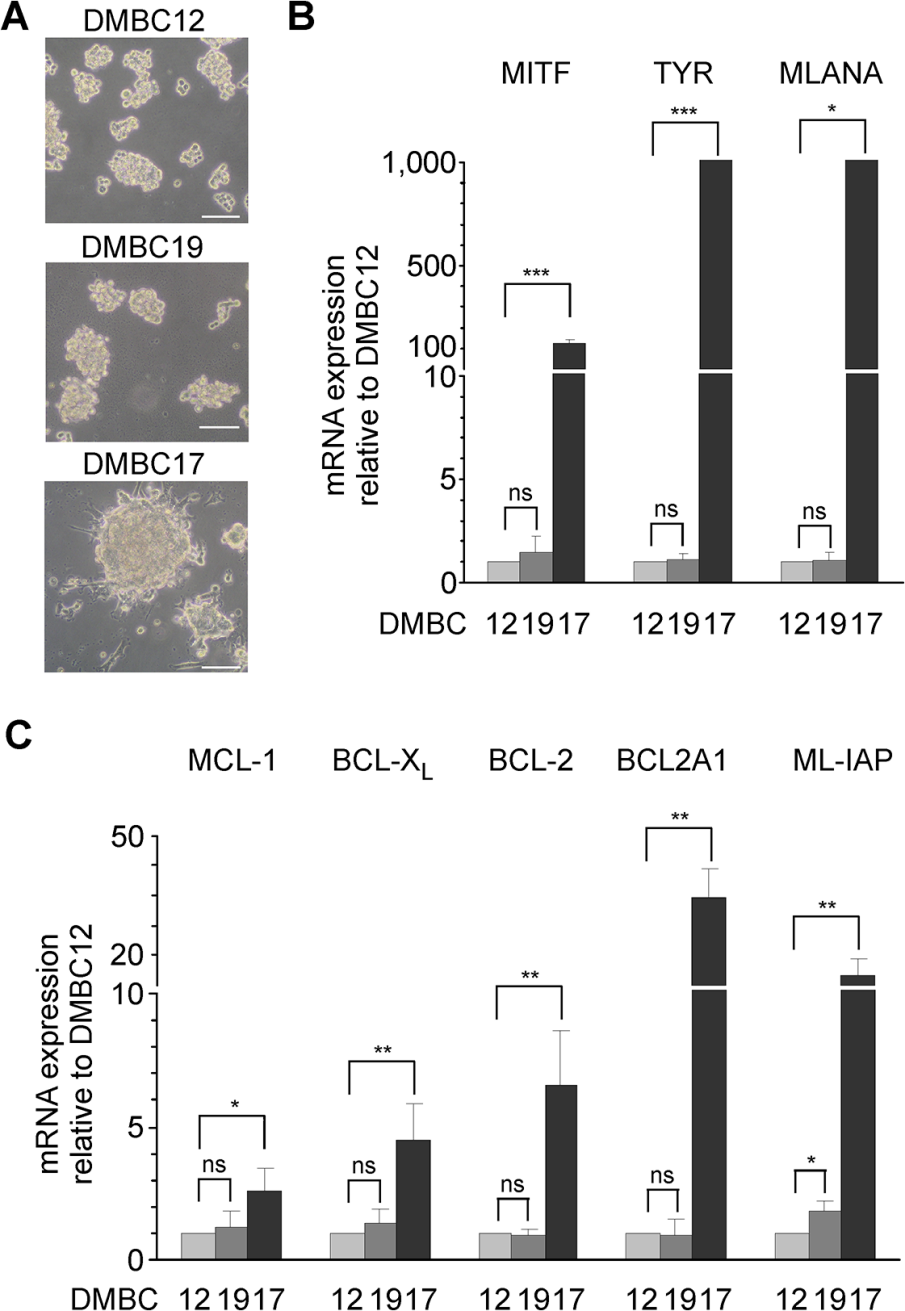
Characteristics of patient-derived cultures used in this study. (A) Morphology of melanoma cells grown in EGF(+)bFGF(+) medium. Fast growing populations DMBC12 and DMBC19 formed cell aggregates, whereas slow growing DMBC17 population formed more dense melanospheres. Scale bar, 100 μm. (B) qRT-PCR was used to assess the expression of MITF and MITF-dependent genes in melanoma cultures in EGF(+)bFGF(+) medium. Data are presented as the means ± SD (n = 4). A fold change more than 1,000 is shown as 1,000. * *p*<0.05; *** *p*<0.001; ns, *p*>0.05. (C) qRT-PCR was used to assess the expression of pro-survival genes in melanoma cells grown in EGF(+)bFGF(+) medium. Data are presented as the means ± SD (n = 4). * *p*<0.05; ** *p*<0.01; ns, *p*>0.05.

We have already shown that different patient-derived melanoma populations grown *in vitro* in EGF(+)bFGF(+) medium exhibit diverse morphology and molecular characteristics regarding immunophenotype and expression of MITF and MITF-dependent genes [18,25,31]. Melanoma populations used in the present study were grown in EGF(+)bFGF(+) medium and formed either small aggregates with weak intercellular connections (DMBC12 and DMBC19), or more dense structures, melanospheres with stronger cell-cell interactions along with adherent counterparts as shown for DMBC17 (Fig 1A). Populations which formed melanospheres had much higher doubling time, which was also observed after they were singularized. For instance, when DMBC17 population was dissociated its doubling time measured within the first 3 days of culturing was estimated as 67 ± 29 hours, whereas for DMBC12 population it was 20 ± 7 hours. DMBC12 and DMBC19 populations had the similar expression of MITF and MITF-dependent genes related to pigmentation, TYR and MLANA, was analyzed (Fig 1B), whereas these genes were expressed at significantly higher levels in the DMBC17 population. When expression of pro-survival BCL-2 genes (MCL-1, BCL-X_L_, BCL-2 and BCL2A1) and ML-IAP from the IAP family was analyzed, there were no substantial differences between DMBC12 and DMBC19 populations (Fig 1C). The expression of pro-survival genes was, however, significantly higher in DMBC17 (Fig 1C). Altogether, these results demonstrate that patient-derived melanoma populations cultured in EGF(+)bFGF(+) medium may markedly differ in expression of MITF and MITF-dependent genes, but also in regard to the baseline pro-survival machinery. We have also compared the expression of pro-survival genes between melanoma populations and normal human adult epidermal melanocytes (NHEM). We have found that MCL-1 was the only pro-survival protein which was overexpressed in all melanoma populations when compared to melanocytes. BCL-X_L_, BCL-2 and ML-IAP were expressed at lower levels in all patient-derived melanoma populations (S1 Fig). The expression of BCL2A1 was significantly higher in MITF^high^ DMBC17 population, but much lower in MITF^low^ DMBC12 and DMBC19 cells compared to melanocytes (S1 Fig).

### Melanoma cells are well protected from death during the early period of adaptation to serum-containing medium

**Fig 2:**
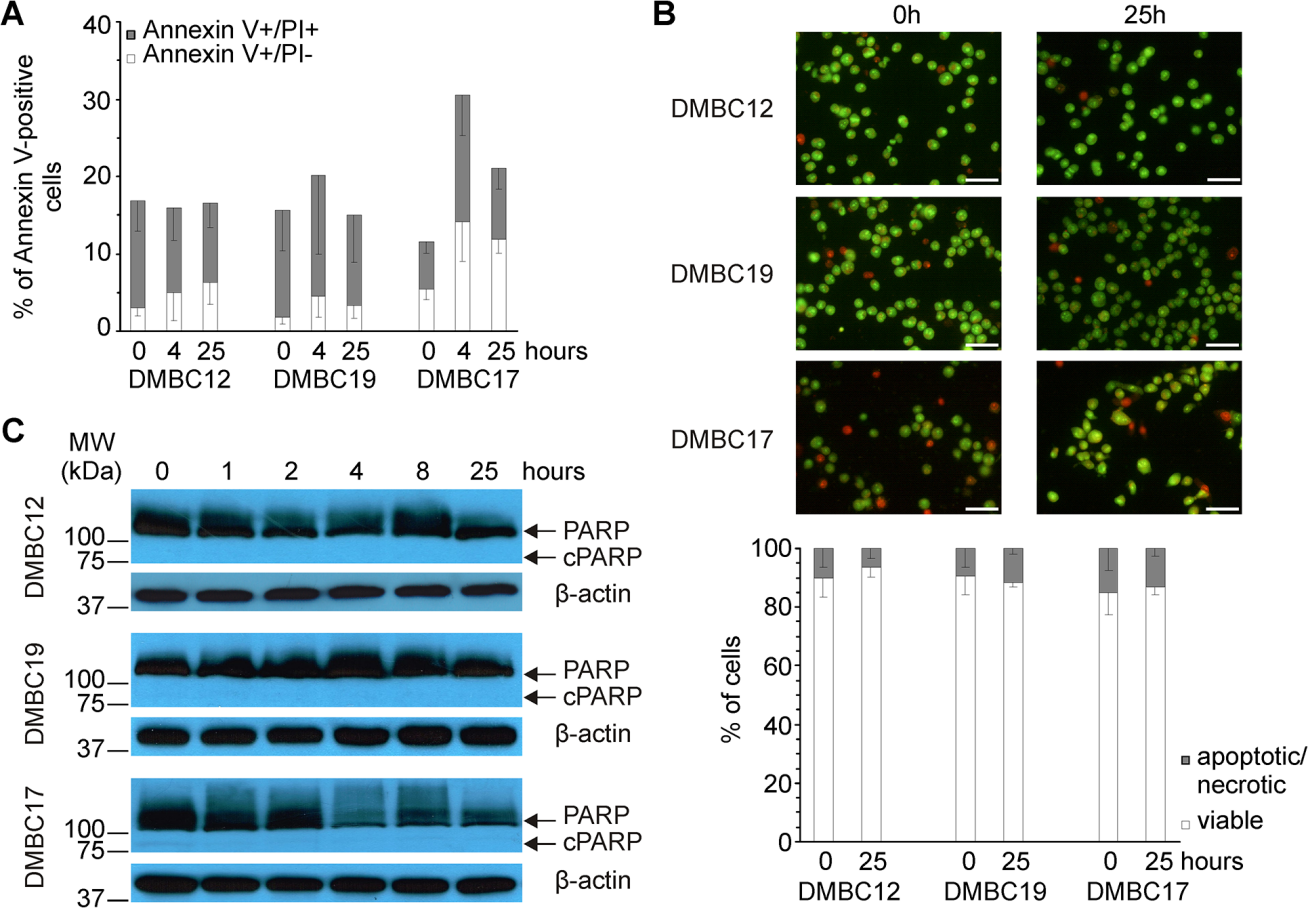
Cell death is not induced in melanoma populations during cell adaptation to serum-containing medium. (A) DMBC12, DMBC19 and DMBC17 populations grown in EGF(+)bFGF(+) medium were dissociated and one day later (0 h) they were exposed to serum-containing medium. Cells were stained with Annexin V/propidium iodide (PI) after 4 h and 25 h, and analyzed by flow cytometry. Data are presented as the means ± SD from three independent experiments. (B) Representative fluorescence microphotographs of melanoma cells stained with acridine orange/ethidium bromide at 0 h and 25 h after medium exchange. Scale bar, 50 μm. Quantitative analysis of the frequency of apoptotic/necrotic and viable cells was shown below. Data are presented as the means ± SD from three independent experiments. (C) Cell lysates were prepared at indicated time points of exposition to serum-containing medium and PARP was immunoblotted (n = 3). Upper and lower arrows indicate full-length and cleaved PARP (cPARP), respectively. Localization and molecular weight (MW) of marker bands are indicated. β-actin was used as a loading control.

Our previous studies have pointed that melanoma populations that were first cultured in EGF(+)bFGF(+) medium and then transferred to serum-containing medium for at least two weeks could be characterized as having reduced self-renewal capacity and heterogeneity and a high invasive potential [25,31]. In the present study, we have addressed the question how a survival machinery is used shortly after a change of microenvironmental conditions. When melanoma cells from EGF(+)bFGF(+) cultures were transferred to serum-containing medium, flow cytometry analysis showed no signs of apoptosis in DMBC12 and DMBC19 populations 4 h and 25 h after medium exchange when compared to control cells (0 h) (Fig 2A). DMBC17 cells seemed to be slightly more sensitive to changes in the microenvironment irrespective of the higher baseline expression of pro-survival genes when compared to DMBC12 and DMBC19 populations (Fig 1C). Microscopic analysis after cell staining with acridine orange and ethidium bromide showed no substantial differences in the frequency of apoptotic/necrotic cells in all melanoma populations before and 25 h after medium exchange (Fig 2B). Accordingly, 89 kDa product of PARP cleavage (cPARP) was not detected in melanoma populations up to 25 h after transfer to serum-containing medium (Fig 2C) indicating that no apoptosis was induced in melanoma cells under these conditions. The immunoreactivity of the antibodies to the cleaved PARP (89 kDa) was confirmed elsewhere [35].

### Immediate response to changes in the microenvironment involves alterations in the pro-survival machinery

**Fig 3:**
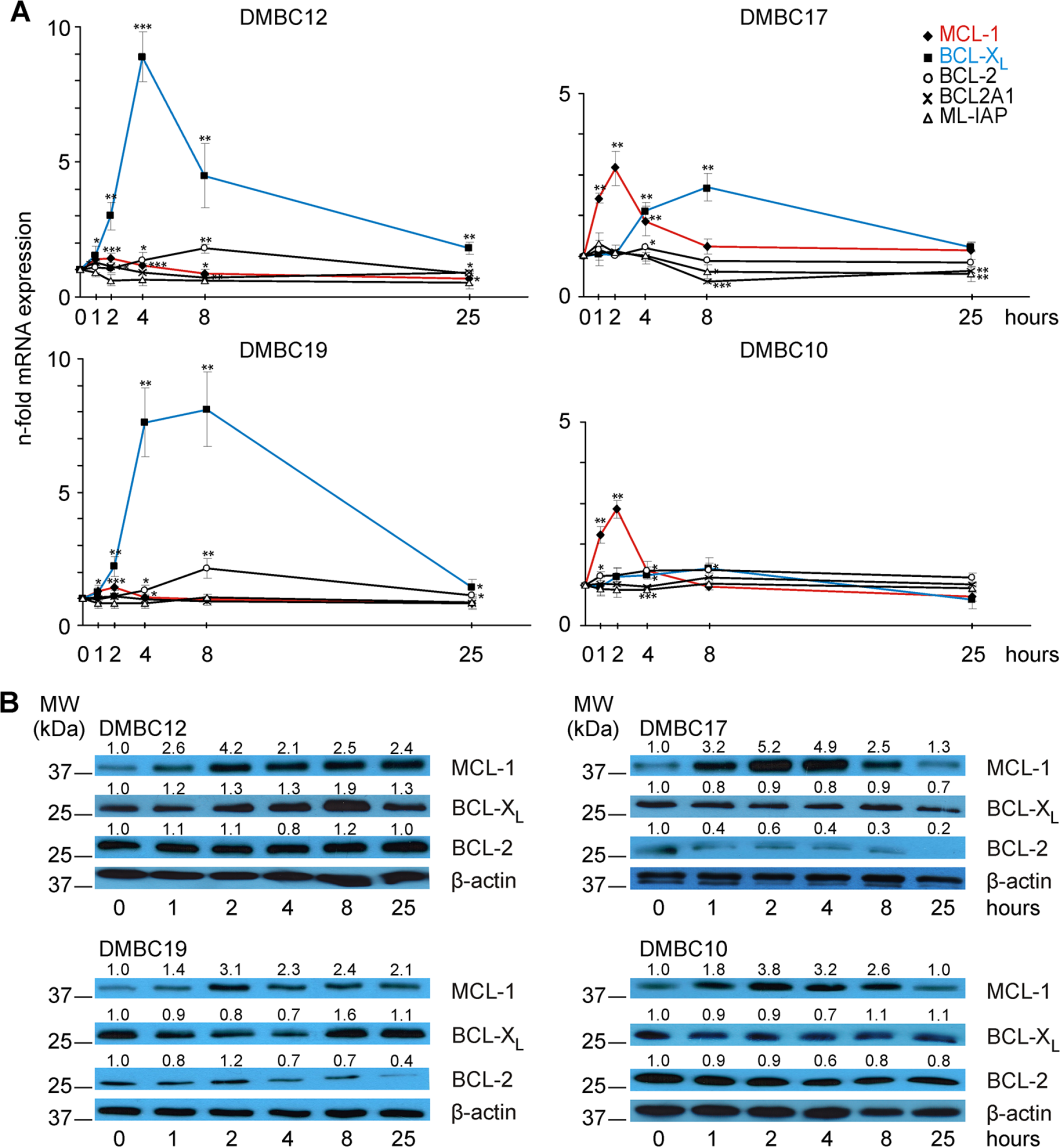
Melanoma cells grown in EGF(+)bFGF(+) medium respond to changes in microenvironment by employing pro-survival machinery. (A) DMBC12, DMBC19, DMBC17 and DMBC10 populations grown in EGF(+)bFGF(+) medium were dissociated and one day later (0 h) they were exposed to serum-containing medium. qRT-PCR was used to analyze the mRNA levels of pro-survival genes at indicated time points of medium exchange. Data are presented as the means ± SD from three independent experiments. * *p*<0.05; ** *p*<0.01; *** *p*<0.001. For clarity, *** (*p*<0.001) for MCL-1 in DMBC12 cells after 1 h was not indicated. (B) Cell lysates were prepared at indicated time points after transfer to serum-containing medium, and pro-survival proteins MCL-1, BCL-X_L_ and BCL-2 were immunoblotted (n = 3). Localization and molecular weight (MW) of marker bands are indicated. β-actin was used as a loading control. Quantification of protein level is shown above the blots.

As melanoma cells cultured in EGF(+)bFGF(+) medium were well protected from cell death during early adaptation to serum-containing medium, we next verified whether this advantage was associated with changes in the pro-survival machinery. The expression of five survival-related genes was analyzed by qRT-PCR after medium exchange. Due to sharp differences in gene expression profiles of DMBC17 cells in comparison to DMBC12 and DMBC19 cells, we extended our study on a previously well characterized population, DMBC10 [25,31]. Concerning gene expression profiles DMBC10 was comparable to DMBC17. Cells from DMBC12 and DMBC19 populations showed a similar pattern of response (Fig 3A). An increase in MCL-1 transcript level was demonstrated peaking 2 h after medium exchange. A more substantial change was observed in BCL-X_L_ mRNA level with the highest increase about 9-fold after 4 h and 8-fold after 8 h in DMBC12 and DMBC19 populations, respectively (Fig 3A). In both populations, BCL-2 mRNA showed a maximum increase after 8 h. The expression levels of BCL2A1 and ML-IAP remained unchanged, or even slightly decreased as demonstrated for ML-IAP in DMBC12 cells. Thus, different pro-survival genes were engaged sequentially during early adaptive response of DMBC12 and DMBC19 cells and an increase in MCL-1 transcript level preceded maximal changes in BCL-X_L_ and BCL-2 expression. DMBC17 and DMBC10 populations differed markedly from DMBC12 and DMBC19 populations in this respect (Fig 3A). First, MCL-1 transcript level augmented more significantly in those cells and this increase was prolonged up to 4 h after transfer to serum-containing medium. Second, changes in BCL-X_L_ mRNA level were not as high as in DMBC12 and DMBC19 populations. The level of transcripts for other anti-apoptotic genes was not substantially changed within 25 h after medium exchange. In all four populations, the early response was transient because observed alterations were attenuated within 25 h of adaptation to serum-containing medium.

We also analyzed changes in the protein level of MCL-1, BCL-X_L_ and BCL-2 as their transcripts showed significant changes in at least two populations during early adaptation to serum-containing medium (Fig 3B). Consistently in all four melanoma populations, MCL-1 protein level increased peaking after 2 h. However, the extent of this response varied between different populations and closely mirrored changes observed at the mRNA level (Fig 3A). Moreover, in DMBC12 and DMBC19 populations, an increase in BCL-X_L_ protein was observed after 8 h (Fig 3B). This was not detected in DMBC10 and DMBC17 populations. The level of BCL-2 protein was not markedly altered during response of melanoma cells to changes in the medium composition. These results indicate that the pro-survival advantage accompanying the adaptation of heterogeneous melanoma populations to different growth conditions may depend on MCL-1 to a variable extent, and BCL-X_L_ may support its function in these populations in which the contribution of MCL-1 is less pronounced.

### Silencing MCL-1 expression sensitizes melanoma cells to death induced by changes in growth conditions

**Fig 4:**
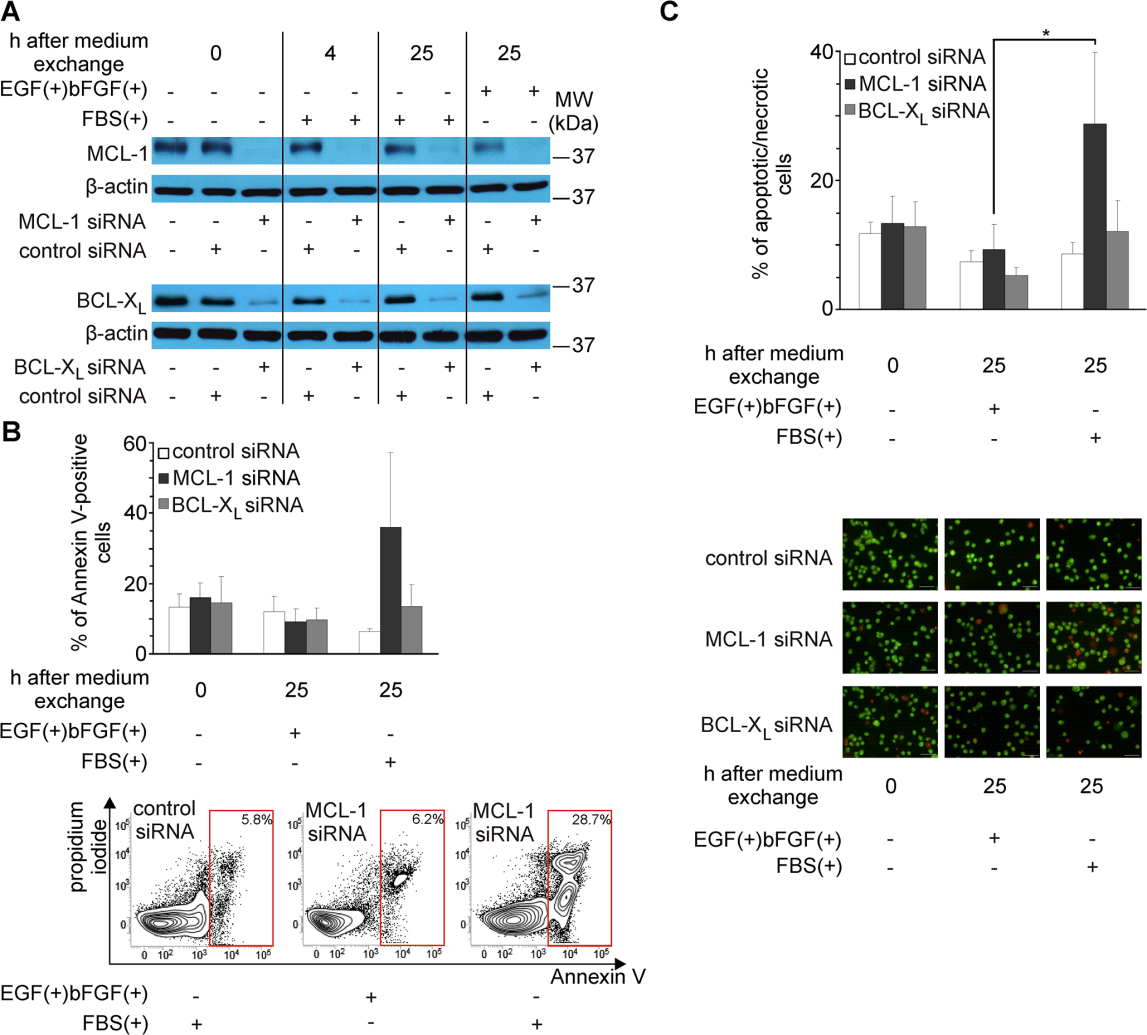
MCL-1 is involved in melanoma cell survival during adaptation to serum-containing medium. DMBC12 cells with silenced expression of MCL-1 or BCL-X_L_ (0h) were exposed to serum-containing medium (FBS(+)), or in parallel they were transferred to fresh EGF(+)bFGF(+) medium. (A) Cell lysates were prepared at indicated time points and MCL-1 and BCL-X_L_ proteins were immunoblotted (n = 3). Localization and molecular weight (MW) of marker bands are indicated. β-actin was used as a loading control. (B) Cells were stained with Annexin V/propidium iodide (PI) before (0h) and 25 h after medium exchange, and analyzed by flow cytometry. The percentage of Annexin V-positive cells is shown. Representative counter plots for melanoma cells after transfection with either control or MCL-1 siRNA are shown. They were acquired 25 h after transfected cell were transferred to either fresh EGF(+)bFGF(+) medium or serum-containing medium (FBS(+)). Data are presented as the means ± SD from three independent experiments. (C) Quantitative analysis of the frequency of melanoma cells stained with acridine orange/ethidium bromide before (0 h) and 25 h after medium exchange. The percentage of apoptotic/necrotic cells is shown. Representative microphotographs are shown below. Data are presented as the means ± SD from three independent experiments. * *p*<0.05.

To validate the functional contribution of MCL-1 and BCL-X_L_ to the protection of melanoma cells from death during early adaptation to serum-containing medium, DMBC12 population was chosen as both MCL-1 and BCL-X_L_ levels were markedly increased in these cells (Fig 3). DMBC12 cells grown in EGF(+)bFGF(+) medium were transiently transfected with siRNA specific for MCL-1 or BCL-X_L_, or control siRNA. The efficiency of silencing was confirmed 16 h after transfection by Western blot for both MCL-1 and BCL-X_L_ (Fig 4A). Flow cytometry and fluorescence microscopy were employed to monitor reduction of cell viability after transfer to serum-containing medium. Transfected cells were exposed in parallel to fresh EGF(+)bFGF(+) medium to exclude cell death induced by medium exchange itself. No effect was observed when DMBC12 melanoma cells with silenced expression of BCL-X_L_ were examined (Fig 4B and 4C). An increase in Annexin V-positive cells (Fig 4B) and apoptotic/necrotic cells (Fig 4C) was obtained when melanoma population with silenced expression of MCL-1 was exposed to serum-containing medium when compared to the same population concomitantly exposed to fresh EGF(+)bFGF(+) medium. This effect is clearly visible in counter plots (Fig 4B) where after silencing MCL-1 the percentage of Annexin V-positive cells, corresponding to early and late phase of apoptosis, was not increased after transfer to fresh EGF(+)bFGF(+) medium, whereas it was raised few times after transfer to serum-containing medium.

### Increase in MCL-1 protein level during response of melanoma cells to serum results from temporarily enhanced stability of MCL-1 transcript

**Fig 5:**
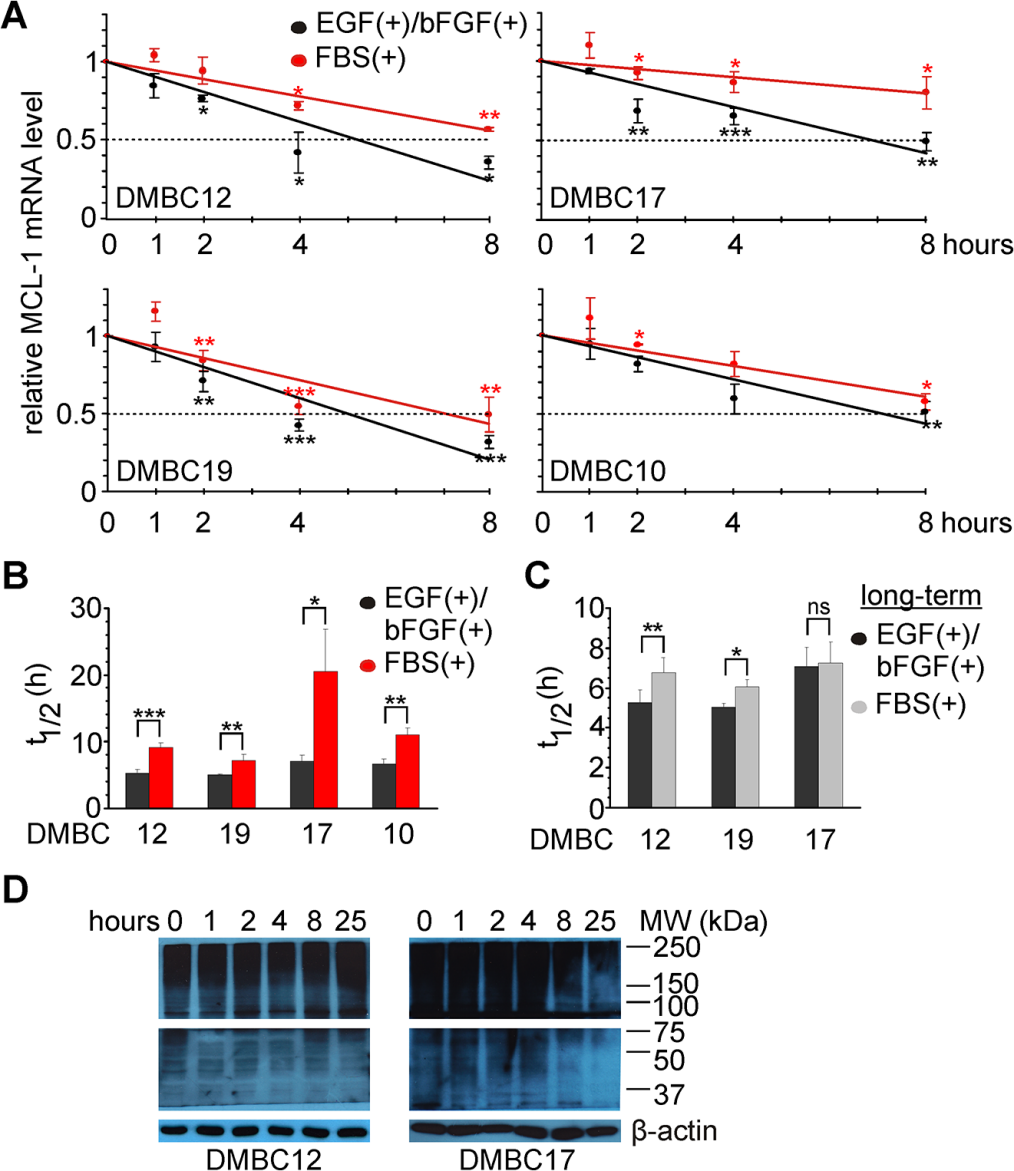
Serum-induced enhanced MCL-1 mRNA stability stands for increased level of MCL-1 protein. (A) Cells were grown in the presence of actinomycin D (2 μg/ml) either in EGF(+)bFGF(+) medium or in serum-containing medium (FBS(+)). qRT-PCR was used to assess MCL-1 transcript level. Data represent the means ± SD from three independent experiments. (B) Decay rates were calculated from the results shown in (A) and expressed as half-lives of MCL-1 transcript (t_1/2_). Data represent the means ± SD from three independent experiments. * *p*<0.05; ** *p*<0.01; *** *p*<0.001. (C) Comparison of MCL-1 mRNA t_1/2_ between long-term melanoma cultures either in EGF(+)bFGF(+) or in serum-containing (FBS(+)) medium. Data represent the means ± SD from three independent experiments. * *p*<0.05; ** *p*<0.01; ns, *p*>0.05. (D) Representative immunoblots showing the accumulation of ubiquitylated proteins at indicated time points of melanoma cell response to serum-containing medium (n = 3). Localization and molecular weight (MW) of marker bands are indicated. β-actin was used as a loading control.

When results of qRT-PCR, immunoblotting and functional assay are concerned, MCL-1 may be considered as an important pro-survival contributor to the immediate response of melanoma cells to serum. Changes in MCL-1 transcript and protein levels correlated well, suggesting an mRNA-based mechanism of regulation. As an increase in MCL-1 transcript was already observed 1 h after serum was introduced, we hypothesized that the stabilization of MCL-1 mRNA might be involved. To test this hypothesis, cells were treated with 2 μg/ml actinomycin D for up to 8 hours to arrest *de novo* RNA synthesis. Longer incubation significantly affected cell viability and was excluded from the analysis. Of note, transcript level of a reference gene *RPS17* was stable during analysis. The level of MCL-1 transcript decreased in a time-dependent manner in all populations incubated with 2 μg/ml actinomycin D (Fig 5A). Decay rates expressed as t_1/2_ for populations grown in EGF(+)bFGF(+) medium were about 5 h for DMBC12 and DMBC19, and about 7 h for DMBC10 and DMBC17 (Fig 5A and 5B). These rates were significantly higher in melanoma cells during adaptation to serum-containing medium (Fig 5A and 5B). They differed between populations and reached about 9 h, 7 h, 21 h and 11 h for DMBC12, DMBC19, DMBC17 and DMBC10 populations, respectively (Fig 5B). Stabilizing effect of the microenvironment on MCL-1 transcript correlated well with the extent of changes in both MCL-1 mRNA and protein levels observed during response to serum-containing medium (Fig 3A and 3B). To check whether this effect was transient we assessed decay rates of MCL-1 mRNA for long-term cultures (two weeks) in serum-containing medium and compared them with those obtained for long-term cultures in EGF(+)bFGF(+) medium (Fig 5C). Stability of mRNA differed between long-term cultures in EGF(+)bFGF(+) and in serum-containing medium for DMBC12 and DMBC19 populations, but no substantial differences were observed for DMBC17 cultured in either medium (Fig 5C). Altogether, these results suggest that the increased stability of MCL-1 transcript is a mechanism used transiently by melanoma cells during their immediate response to changes in the microenvironment. In addition, long-term growth in serum-containing medium slightly increases stability of MCL-1 transcript in fast cycling populations, DMBC12 and DMB19, when compared with stability measured in EGF(+)bFGF(+) medium.

When the accumulation of ubiquitylated proteins was analyzed as a readout of the overall protein turnover, no differences were found during response of melanoma cells to serum-containing medium, both in populations less and more dependent on MCL-1 as demonstrated for DMBC12 and DMBC17, respectively (Fig 5D). These results suggest that changes in MCL-1 protein are predominantly driven by enhanced MCL-1 transcript stability.

### Immediate response to changes in the microenvironment involves alterations in essential melanoma-related signal transducers

**Fig 6:**
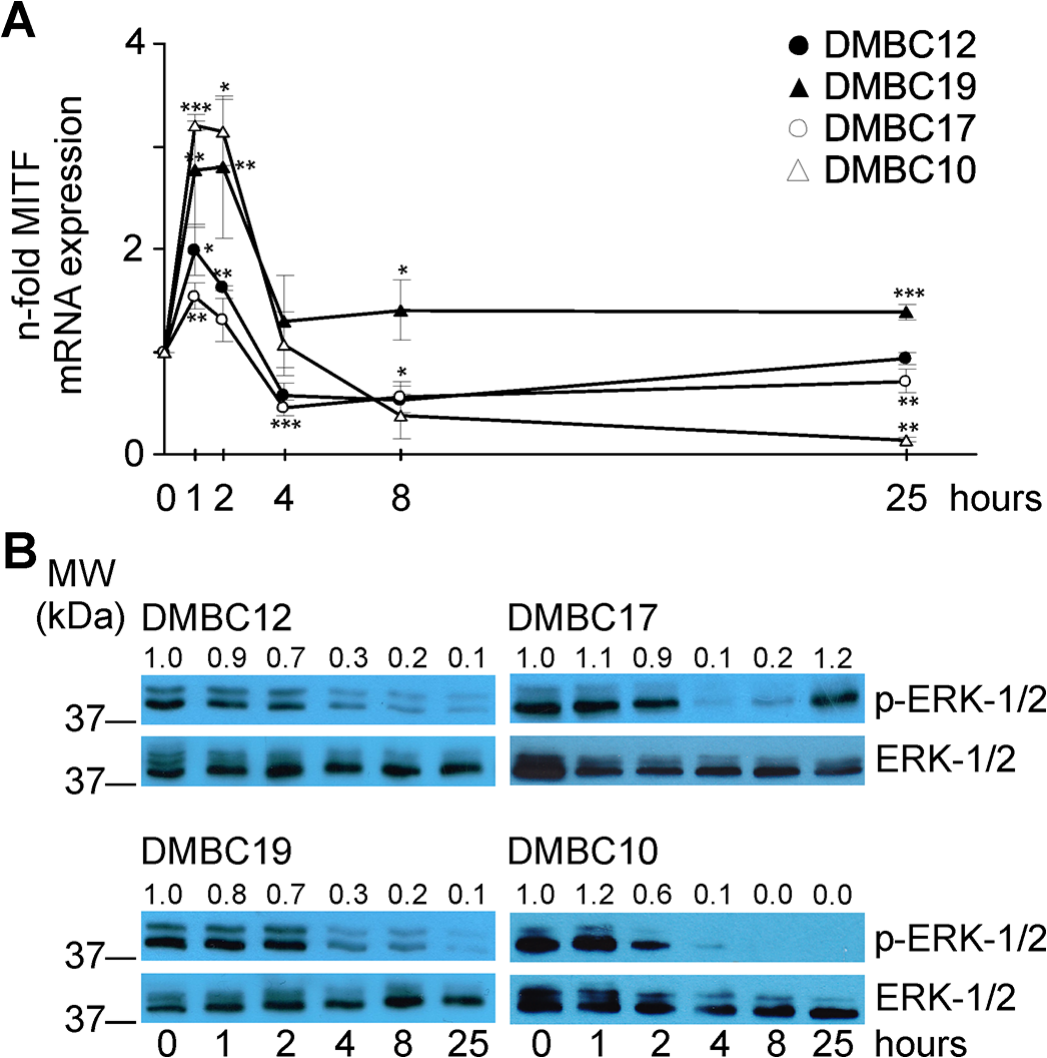
Immediate response to changes in microenvironment involves alterations in MITF level and ERK-1/2 activity. (A) Level of MITF mRNA was analyzed at indicated time points during early adaptation period of melanoma cells to serum-containing medium. Data are presented as the means ± SD from three independent experiments. * *p*<0.05; ** *p*<0.01; *** *p*<0.001. (B) Immunodetection of phosphorylated ERK-1/2^Thr202/Tyr204^ (p-ERK-1/2) was used a readout of their activity (n = 3). Localization and molecular weight (MW) of marker bands are indicated. ERK-1/2 was used as a loading control. Quantification of p-ERK-1/2 level is shown above the blots.

We have also checked whether MITF and ERK-1/2 were involved in adaptive melanoma cell response as both MITF expression and ERK-1/2 activity clearly differ between cells continuously cultured in either of tested medium [25]. In all populations, MITF mRNA level was significantly increased 1–2 h after cells were exposed to serum-containing medium, and then substantially decreased. That reduction was well pronounced in the populations with high baseline MITF transcript level (DMBC10 and DMBC17) (Fig 6A). When the activity of ERK-1/2 kinases was analyzed, a time-dependent attenuation of the level of phosphorylated ERK-1/2 was detected in all populations (Fig 6B). ERK-1/2 activity in DMBC17 cells was already restored 25 h after transfer to serum-containing medium. Altogether, these results indicate that melanoma cells may engage a complex adaptive response upon changes in microenvironment involving alterations in specific pro-survival molecules and critical regulators of melanoma biology.

## Discussion

Several *in vitro* experimental models are used to study melanoma biology and cell response to drugs but the most broadly applied is the monolayer of cells grown in serum-containing medium. Those well-established settings have, however, several disadvantages and reduced heterogeneity, lack of three-dimensional structure, disabled cell-cell contacts and formation of extracellular matrix might be mentioned among others. We have shown recently that melanoma cells directly derived from surgical specimens and grown in bFGF(+)EGF(+) stem cell medium as anchorage-independent melanospheres more closely resemble the original tumors than do monolayers maintained in the presence of serum [18,25]. The observed changes in the transcriptomes between cells grown in either medium were reflected by alterations in the frequencies of melanoma cells that exerted diverse functions, including self-renewal, differentiation, proliferation and invasiveness. Culturing of melanoma cells in serum-containing medium enriched melanoma populations in cells with invasive phenotype [25]. More cells grown in this medium were capable of invading Matrigel and transcriptome profiling showed the elevated expression of several genes encoding proteins involved in invasiveness including DNA binding protein ID3, non-histone chromatin binding protein HMGA2, matrix metalloproteinases MMP9 and MMP2, the adhesion molecule MCAM and S100 calcium binding protein A4 (S100A4) [25]. Survival in the circulation is a critical step of metastasis [37]. That microenvironment eliminates melanoma cells with non-permissive phenotypic state [38]. We assumed that serum-containing medium might mimic to a certain extent the microenvironment the cells are exposed to during hematogenous dissemination, and this is why the outgrowth of subpopulations with high invasiveness was observed when cells from melanospheres were transferred to serum-containing medium [25]. Recently, induction of monolayers by serum from anchorage-independent multicellular spheroids was considered as a good *in vitro* model of hepatocellularcarcinoma metastasis [39]. In the present study, we concentrated exclusively on early pro-survival response of melanoma cells to changes in the microenvironment, namely to the replacement of bFGF and EGF with serum. We took advantage from having patient-derived heterogeneous melanoma populations mimicking well the parent tumors that unlike established cell lines were never before exposed to serum-containing medium. We have found that: (1) melanoma cells are well protected from cell death during early period of adaptation to serum-containing medium; (2) MCL-1 and BCL-X_L_ are implicated in adaptive response to new growth conditions; (3) increased MCL-1 mRNA and protein levels in response to serum result from transiently enhanced stability of MCL-1 transcript; (4) immediate response involves alterations in essential melanoma-related signal transducers MITF and ERK1/2; (5) the response to serum differs between populations with distinct morphology and baseline expression of pro-survival genes.

Melanoma cells are well equipped with anti-apoptotic mechanisms that support cancer-related events, including metastasis [40]. The microenvironment can modulate the activity of the pro-survival machinery, and the regulation of MCL-1 and BCL-X_L_ expression by microenvironment-derived factors has been already reported. *MCL1* contains serum response elements (SREs) in its promoter that may be activated in response to a variety of mitogenic and stress stimuli [41]. Both BCL-X_L_ [42,43] and MCL-1 [44,45] have been also implicated in the cell response to serum starvation. It has been shown that MCL-1 and BCL-X_L_ expression can be triggered by granulocyte-macrophage colony stimulating factor (GM-CSF) and interleukin-3 (IL-3) in an erythroleukemic cell line [46]. In prostate cancer cells, platelet-derived growth factor (PDGF) has been shown to induce MCL-1 expression [47]. Insulin-like growth factor-binding proteins (IGFBPs) derived from carcinoma-associated fibroblasts (CAFs) stabilized MCL-1 protein to protect breast cancer cells from cell detachment-induced death (*anoikis*) [48]. Both *anoikis* and endoplasmic reticulum (ER) stress occur during tumor cell dissemination [49] making the formation of metastases a highly inefficient process [50]. In this respect, overexpression of BCL-X_L_ has been already linked to survival advantage in circulating breast cancer cells [51] that is in line with *anoikis*-preventing up-regulation of BCL-X_L_ by chemokine (C-C motif) ligand 19 (CCL19) demonstrated in this type of tumor cells [52]. In glioma cells, short-term effects of BCL-X_L_ upregulation were related to cell survival, but in the long run BCL-X_L_ contributed to their pro-invasive potential [53]. In several cell lines including those derived from melanoma, BCL-X_L_ has been implicated in the pro-angiogenic phenotype of cancer cells by regulation of CXCL8 expression [54]. Survival of melanoma cells after cell detachment may depend on MCL-1 [55]. MCL-1 is also an adaptive contributor protecting melanoma cells from the ER stress-induced cell death [56,57]. Thus, MCL-1 and BCL-X_L_ expression may be affected by fluctuating extracellular signals from the microenvironment.

A pro-survival potency of BCL-2-like proteins towards exogenous stressors may be influenced by their stability [58]. An inhibitory effect of the microenvironment on the transcript decay under stress conditions has been already reported [59]. Both MCL-1 transcript and protein are dynamically regulated in the cell and have been consistently defined as unstable and rapidly degraded [58,60–64], also in response to metabolic stress [65]. MCL-1 half-life can be prolonged, however, upon ERK-1/2-dependent phosphorylation of MCL-1 at Thr^163^ [66,67], and this effect has been also reported in melanoma cells [68]. ERK-1/2-dependent regulation of MCL-1 level and stability has been already demonstrated in hematopoietic cells in the context of immediate response to cytokine stimulation [46]. In the present study, we have demonstrated that an increase in MCL-1 protein level in melanoma cells immediately after transfer to serum-containing medium results from transiently increased MCL-1 mRNA stability as the extent of changes in MCL-1 mRNA and protein levels well portrayed the extent of the MCL-1 transcript stabilization. In addition, we have not reported changes in the overall protein turnover, and the association between ERK-1/2 activity and MCL-1 level was not a part of an immediate response of melanoma cells to serum because serum-induced inhibition of ERK-1/2 did not coincide with the reduction of MCL-1 at the protein level. By using actinomycin D-based approach, we were unable to estimate BCL-X_L_ transcript decay rate that is in line with the data showing that the half-lives of both BCL-X_L_ mRNA and protein are much longer than those of MCL-1 [58,60].

Adaptive response of melanoma cells may involve not only critical changes in the anti-apoptotic machinery. MITF, a melanocyte-specific modulator also recognized as a lineage addiction oncogene in melanoma, has been described as regulating expression of anti-apoptotic and stress-attenuating genes in melanocytes and melanoma cells [26]. Recently, we have reported MITF as an essential regulator of microenvironment-driven alterations in melanoma phenotype [25]. In the present study, we have shown that MITF level was already changed at early stages of adaptation to serum-containing medium. This corresponds well to studies that directly implicated MITF in phenotype switching of melanoma cells [22,24]. We have also demonstrated reduced activity of ERK-1/2 immediately after transfer to serum-containing medium. This effect, however, was transient as established long-term cultures in serum-containing medium are characterized by higher activity of ERK-1/2 than populations grown in EGF(+)bFGF(+) medium [21,25]. Despite the multiplicity of cellular processes regulated by these kinases, they are also recognized as regulators of cell cycle progression [69]. It has been evidenced that stress signals derived from a foreign microenvironment may evoke low intracellular ERK-1/2/p38 signaling ratio that is indicative of a growth arrest, and increase in ERK-1/2 activity stands for active melanoma cell proliferation [70]. Consistently, we have already documented a transient attenuation of proliferation rate during first two days of melanoma cell adaptation to serum-containing medium [31]. Thus, our previous [25] and present studies indicate that modulation of MITF level and ERK-1/2 activity may be implicated in response to foreign microenvironment, also in other processes than cell survival.

We have demonstrated that the response to changes in the microenvironment varies between melanoma populations derived from different surgical specimens. Those melanomas in which microenvironment-driven changes in MCL-1 level were not substantial involved an increase in BCL-X_L_ expression as a complementary alteration. MCL-1 dependence seems to be especially important in melanoma populations with high MITF level and activity, whereas in populations in which MITF is expressed at low levels, BCL-X_L_ may be sequentially involved after MCL-1 expression is reduced. We have shown that MITF level and activity correlates well with the extent of heterogeneity of patient-derived melanoma cultures in EGF(+)bFGF(+) medium [18,25]. Thus, the character of immediate pro-survival response of melanoma cells seems to be also cell-context dependent. In contrast to a study on the established melanoma cell lines [71], we have demonstrated that MCL-1 inhibition by specific siRNA did not cause significant cell death in patient-derived melanoma populations. Importantly, despite efficient silencing of MCL-1, cell death was not massive indicating that MCL-1 may be only critical for the survival of a specific subpopulation in the context of adaptation to serum.

Our present study strongly emphasizes the need for investigating early adaptive responses of melanoma in an experimental model preserving the original tumor characteristics. Study on glioblastoma cells has indicated that several metabolites were transiently regulated by hypoxic stress [72]. By employing a model mimicking organs of breast cancer metastasis, it has been evidenced that several gene clusters associated with cell adaptation are transiently activated when tumor cells meet a foreign microenvironment [73]. Pro-survival molecules were shown to be involved in the microenvironment-induced response of hematological cancer as shown for MCL-1-mediated survival of CLL cells in bone marrow stromal microenvironment [74]. Thus, deciphering the contextual dependency on a specific pro-survival protein may hold strong implications for eradicating cancer cells [75]. Microenvironment-driven phenotype switch of melanoma cells may hold critical implications not only for metastasis, but also for therapy resistance [12]. It can be exemplified for MAPK pathway inhibition [76,77]. Targeting BCL-2-like proteins with BH3 mimetics improved the efficiency of BRAF^V600E^ inhibition as well as reduced acquired resistance of melanoma cells to this strategy [78]. Therefore, targeting of BCL-2-like proteins is an interesting option in anticancer therapy [68,79,80], and our study points to the involvement of MCL-1, especially in MITF^high^ populations, in phenotypic plasticity of melanoma cells used in immediate adaptation to modifications of the microenvironment.

## Supporting Information

S1 Fig Expression of pro-survival genes in melanocytes and melanoma populations. qRT-PCR was used to assess the expression of pro-survival genes in tested melanoma cells *versus* melanocytes (NHEM) grown as described in Materials and Methods. Data are presented as the means ± SD (n = 4). * *p*<0.05; ** *p*<0.01; *** *p*<0.001.
(TIF)

S1 Table Primer sequences, forward (F) and reverse (R) used in the qRT-PCR experiments. (DOC)
