# Supplementary figures and images for: MCL-1, BCL-XL and MITF Are Diversely Employed in Adaptive Response of Melanoma Cells to Changes in Microenvironment
Source: PLoS One. 2015 Jun 2;10(6):e0128796. doi: 10.1371/journal.pone.0128796 (PMC4452715; doi:10.1371/journal.pone.0128796)

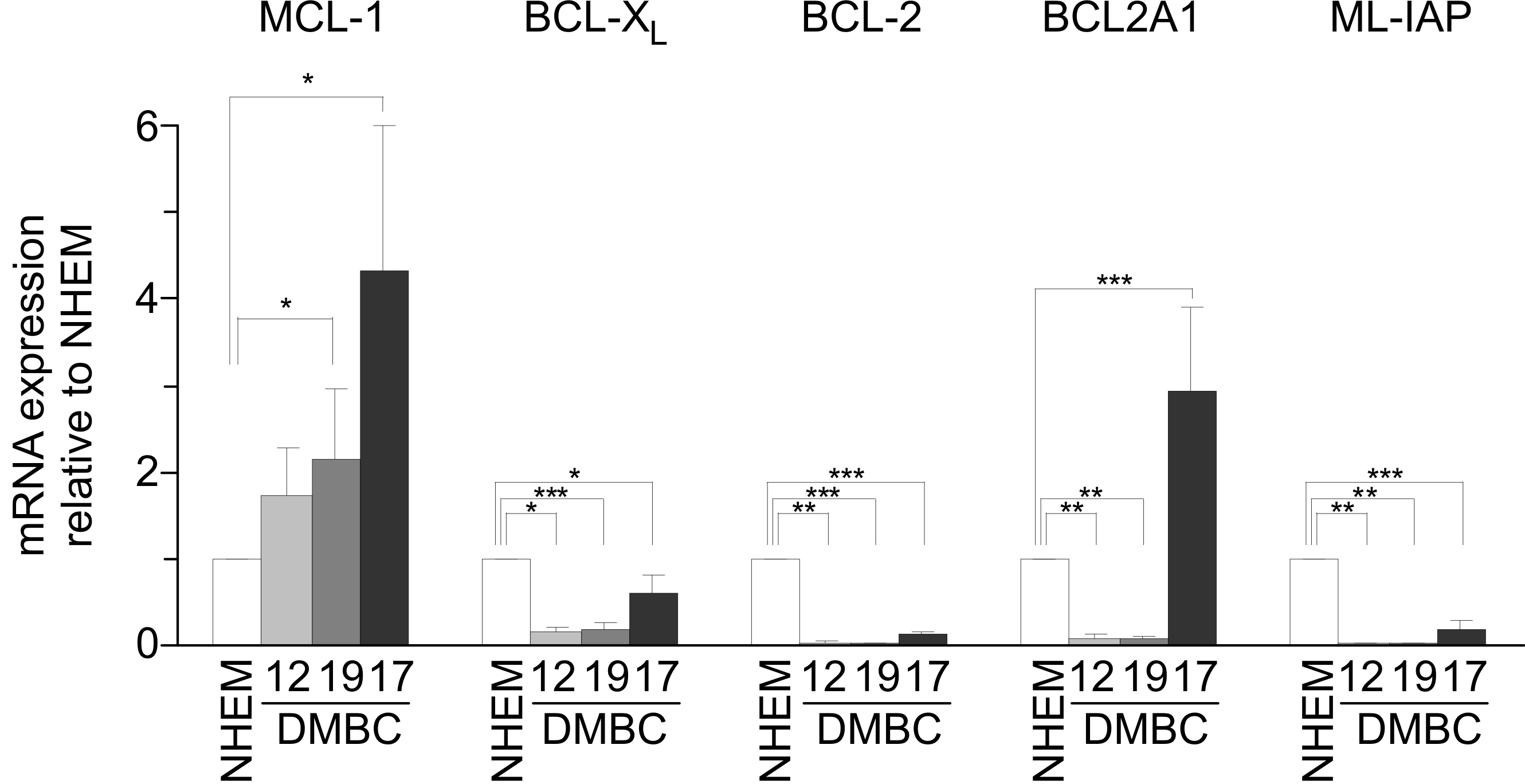

Supplement: S1 Fig — qRT-PCR was used to assess the expression of pro-survival genes in tested melanoma cells versus melanocytes (NHEM) grown as described in Materials and Methods. Data are presented as the means ± SD (n = 4). * p<0.05; ** p<0.01; *** p<0.001. (TIF) [file pone.0128796.s001.tif]
