# Supplementary material for: MCL-1, BCL-XL and MITF Are Diversely Employed in Adaptive Response of Melanoma Cells to Changes in Microenvironment
Source: PLoS One. 2015 Jun 2;10(6):e0128796. doi: 10.1371/journal.pone.0128796 (PMC4452715; doi:10.1371/journal.pone.0128796)
Supplement: S1 Table — (DOC) [file pone.0128796.s002.doc]

| Gene | Sequence | T_M_ (^o^C) | Amplicon (bp) |
| --- | --- | --- | --- |
| *BCL2A1* | F: GGATAAGGCAAAACGGAGGCTG  R: CAGTATTGCTTCAGGAGAGATAGC | 62  59 | 183 |
| *BCLXL* | F: GGCGGATTTGAATCTCTTTCTC  R: TTATAATAGGGATGGGCTCAACC | 60  61 | 141 |
| *BCL2* | F: ATGTGTGTGGAGAGCGTCAA  R: CCGGTTCAGGTACTCAGTCA | 58  60 | 81 |
| *BIRC7* | F: TGTCCACAGTGTGCAGGAGACT  R: GGCACTTTCAGACTGGACCTCCT | 64  64 | 127 |
| *MCL1* | F: ATTAGATATGCCAAACCAGCT  R: CTTCCAAGGATGGGTTTGTG | 58  58 | 118 |
| *MITF* | F: ACCGTCTCTCACTGGATTGG  R: TACTTGGTGGGGTTTTCGAG | 60  58 | 104 |
| *MLANA* | F: GGACAGCAAAGTGTCTCTTCAAG  R: TCAGGTGTCTCGCTGGCTCTTA | 63  64 | 132 |
| *RPS17* | F: AATCTCCTGATCCAAGGCTG  R: CAAGATAGCAGGTTATGTCACG | 60  58 | 142 |
| *TYR* | F: CTGGAAGGATTTGCTAGTCCAC  R: CCTGTACCTGGGACATTGTTC | 62  61 | 106 |

**S1 Table.** Primer sequences, forward (F) and reverse (R) used in the qRT-PCR experiments.
